# Post-fire recovery in fire-sensitive tropical forests: the role of habitat loss and resilience thresholds

**DOI:** 10.1007/s00442-025-05856-4

**Published:** 2026-01-06

**Authors:** Tamiris Cantelli Sardinha, Lorenzo De Melo Nogues Giampaolo, Ederson Godoy, Bruno F. C. B. Adorno, Bianca Dinis, Wellington Corrêa, Vinícius Munhoz Barbosa, Lucas Andrigo Maure, Augusto João Piratelli, Milton Cezar Ribeiro, Érica Hasui

**Affiliations:** 1 Instituto de Ciências da Natureza, Universidade Federal de Alfenas, Alfenas, Brazil; 2 Departamento de Ecologia e Recursos Naturais, Universidade Federal de São Carlos, São Carlos, Brazil; 3 Earth Observation and Geoinformatics Division, National Institute for Space Research (INPE), São José Dos Campos, São Paulo Brazil; 4 Departamento de Ciências Ambientais/CCTS, Universidade Federal de São Carlos, Sorocaba, SP Brazil; 5 Laboratório de Ecologia Espacial e Conservação (LEEC), Departamento de Ecologia, Instituto de Biociências, Universidade Estadual Paulista—UNESP, Rio Claro, SP Brazil; 6 Centro de Estudos Ambientais (CEA), Universidade Estadual Paulista—UNESP, Rio Claro, SP Brazil

**Keywords:** Atlantic forest, Avian, Disturbance, Forest cover, Landscape, Wildfire

## Abstract

Fire is a major driver of ecological change in tropical forests, yet the combined effects of fire history and habitat loss on frugivorous birds and post-fire vegetation recovery remain poorly understood. We evaluated how fire severity, extent, and frequency—together with forest cover—shape frugivore abundance and influence short-term (≤ 10 years) forest resilience in the Cantareira–Mantiqueira Corridor, southeastern Brazil. Using frugivore surveys conducted at fixed points across 15 landscapes, combined with satellite-derived fire metrics and kNDVI-based estimates of engineering resilience, we applied a set of hypothesis-driven Generalized Linear Models and segmented regressions. Fire characteristics alone did not explain frugivore abundance; however, a strong interaction between fire severity and forest cover revealed that forest cover buffered fire impacts under a low severity but failed under high-severity conditions, leading to marked declines in frugivore abundance. Forest recovery was primarily governed by fire characteristics rather than by frugivore abundance. Fire extent showed a linear negative effect on kNDVI trend, while fire frequency displayed nonlinear behavior, with a threshold near three fire events beyond which recovery became highly variable and unpredictable. Together, these findings provide evidence that fire and habitat loss interact to shape frugivore communities, and that repeated burning can push fire-sensitive tropical forests toward resilience thresholds. Effective conservation and restoration strategies must therefore integrate both fire prevention and habitat maintenance to safeguard ecosystem recovery.

**Graphical abstract:**

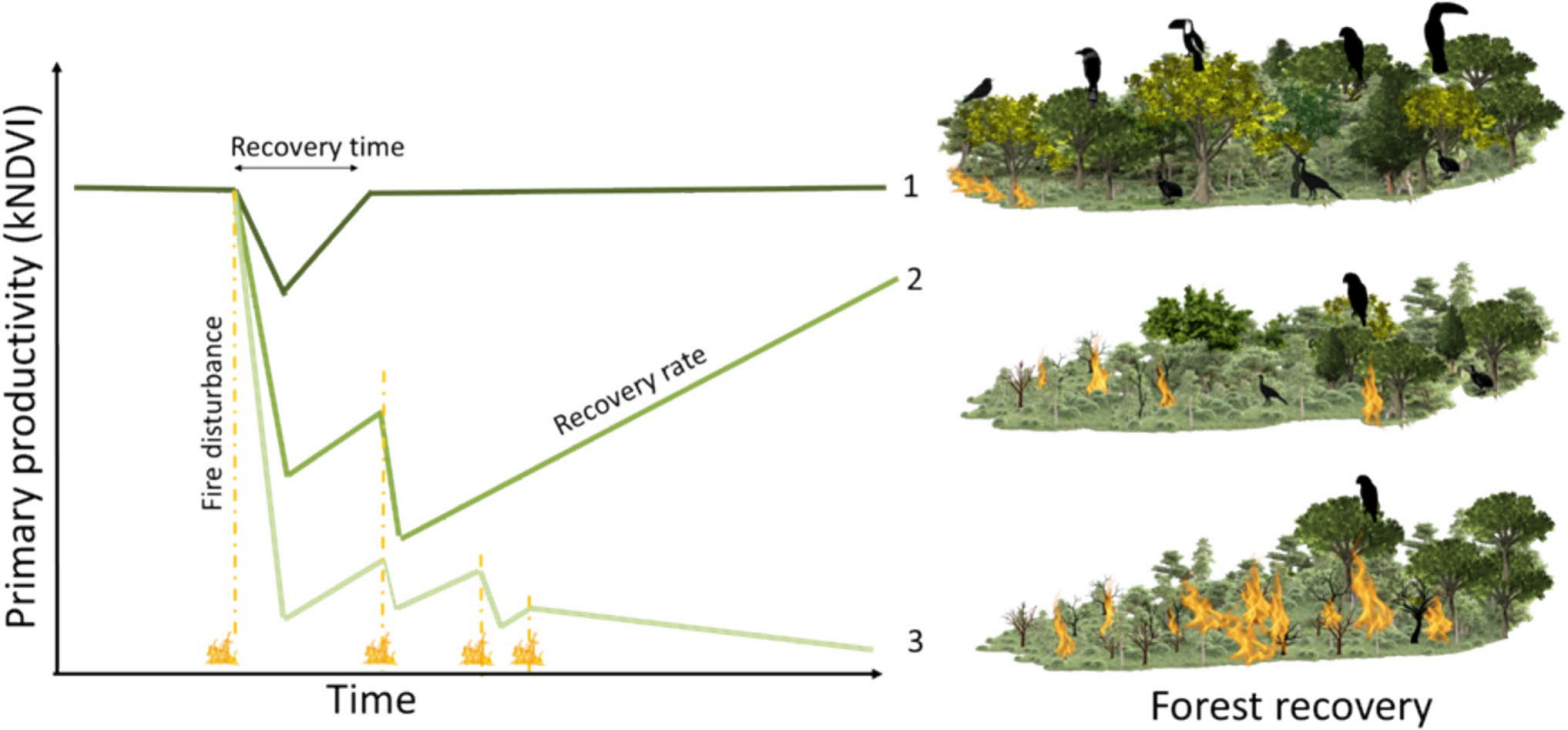

**Supplementary Information:**

The online version contains supplementary material available at 10.1007/s00442-025-05856-4.

## Introduction

Climate change and human activities have increasingly altered fire characteristics, leading to significant changes in vegetation structure and species composition (Adams 2013; Cochrane 2003). Tropical forests are particularly vulnerable to fire due to the lack of adaptive traits in native species, making the escalating size and frequency of wildfires especially concerning. Projections indicate that fire frequency may rise by 44% in the Amazon, 80% in the Congo, and 123% in Indonesia, primarily due to deforestation and climate variability (Wimberly et al. 2022, 2023). Additionally, reduced precipitation and increased wind speeds are expected to escalate fire risks by lowering fuel moisture and facilitating the rapid spread of flames, resulting in larger and more severe fires (Hoffmann et al. 2003).

These fire disturbances play a crucial role in shaping the dynamics of secondary forest succession, influencing both the rate and trajectory of vegetation recovery (Chazdon et al. 2007). The ability of an ecosystem to return to its pre-disturbance state depends on fire severity, environmental conditions, and biological legacies, such as seed dispersers, seed banks, and resprouting capacity (Cramer et al. 2008). When essential physical and biological conditions are preserved, the system can gradually regain its pre-disturbance structure and function. However, high fire severity or recurrent fires can deplete residual vegetation and seed sources, slowing forest regrowth and biomass accumulation. Under extreme conditions, successional processes may become arrested or lead to prolonged periods of degraded states, where ecosystem recovery is highly uncertain (Chazdon et al. 2007; Standish et al. 2014).

The interaction between fire and habitat fragmentation further complicates these recovery dynamics. Fragmented forests, characterized by increased edge effects and reduced connectivity, are more susceptible to fire incidents. Edges created by fragmentation can lead to microclimatic changes, such as increased temperature and decreased humidity, making forests more prone to fires (Driscoll et al. 2021). This synergistic interaction between fire and fragmentation can reduce the rate of vegetation recovery, prolonging the time required for forests to return to their pre-disturbance state (Cochrane 2001).

Predicting recovery trajectories remains challenging due to the interplay of stochastic and deterministic factors that influence ecosystem resilience (Arroyo-Rodríguez et al. 2017; Standish et al. 2014). Nonlinear patterns in fire effects may indicate thresholds beyond which forest recovery becomes increasingly unpredictable, raising the risk of persistent degradation (Cumming et al. 2012; Simmonds et al. 2019). Identifying these thresholds is crucial for determining whether post-fire forests can recover autonomously or require active restoration interventions (Johnstone et al. 2016; Pausas and Keeley 2014).

Engineering resilience, as applied here, refers to the capacity of a system to regain its original state after disturbance, focusing on the speed and direction of recovery rather than the existence of alternative stable states (Holling 1996). This concept assumes that ecosystems have a reference condition and that recovery can be measured through temporal trends in key indicators. In the context of post-fire vegetation dynamics, engineering resilience is shaped by environmental conditions (climate, soil properties, topography), biological attributes (species composition, functional groups), and fire characteristics (frequency, extent, and severity), as well as landscape structure (Chambers et al. 2019; Nikinmaa et al. 2020).

At the landscape level, spatial configuration, connectivity, and proximity to unburned or low-severity patches play a fundamental role in forest resilience. Landscape structure influences seed disperser movements, facilitating the transfer of seeds from surviving vegetation to burned forests, thereby enhancing regeneration potential (Carlo et al. 2013; Chazdon 2003). However, in fragmented landscapes, reduced connectivity can limit seed dispersal and slow recovery, as the movement of dispersers is constrained, and the influx of propagules is diminished (Aben et al. 2012; Astudillo et al. 2019; Molina et al. 2023; Sultaire et al. 2023). Despite its critical role, most studies on forest recovery focus on local site conditions, overlooking the broader influence of landscape structure on post-fire regeneration (Arroyo-Rodrıguez et al. 2017).

Beyond landscape connectivity, the resilience of regenerating forest patches is influenced by intrinsic factors, such as seed rain, seed banks, seedling recruitment, and soil conditions, as well as their interaction with broader spatial dynamics (Molina et al. 2023; Pulla et al. 2015). Frugivorous birds play a key role in these processes by dispersing seeds across post-fire landscapes, potentially accelerating regeneration. However, the extent to which fire characteristics, habitat loss, and fragmentation influence frugivore abundance—and consequently seed dispersal—remains inadequately understood (Arroyo-Rodrıguez et al. 2017). Additionally, fire characteristics such as frequency and severity are often overlooked in ecological assessments, limiting our understanding of their role in shaping species responses and ecosystem resilience (Giorgis et al. 2021). Explicitly examining the interactions between fire regimes, frugivore-mediated seed dispersal, and forest regeneration in fragmented landscapes is crucial for designing effective conservation and restoration strategies (Driscoll et al. 2021).

Addressing these knowledge gaps is essential for developing effective conservation strategies in fire-susceptible landscapes. Understanding fire thresholds is crucial for determining whether active restoration efforts are necessary or if natural recovery processes can proceed without intervention. Identifying these thresholds helps guide management actions, ensuring that resources are allocated efficiently while minimizing long-term ecological degradation.

Thus, this study aims to evaluate how fire severity, extent, and frequency—together with forest cover—shape frugivore abundance and influence short-term (≤ 10 years) forest resilience. By elucidating these relationships, we aim to answer the following research questions and test the associated hypotheses and predictions:

Q1. How do fire characteristics (extent, severity, frequency, and time since fire) influence the abundance of frugivorous birds?

- Hypothesis: Fire characteristics affect frugivore populations by altering habitat structure and resource availability (Barlow and Peres 2004a, b; Cochrane and Schulze 1999; Driscoll et al. 2021; Robinson et al. 2013).
- Prediction: Frugivore abundance decreases as fire disturbance and habitat loss intensity increase (Jones et al. 2023).

Q2. Does fire interact with habitat loss to negatively affect frugivore abundance?

- Hypothesis: Fire interacts with habitat loss, leading to a greater decline in frugivore abundance than either factor alone (Coop et al. 2019; Driscoll et al. 2021; Marjakangas et al. 2020; Nitschke and Innes 2006).
- Prediction: Frugivore abundance decreases as both fire disturbance and habitat loss intensity increase.

Q3. Does forest recovery decline nonlinearly beyond a critical fire threshold, leading to prolonged degradation?

- Hypothesis: The rate of forest recovery declines nonlinearly beyond a critical fire threshold, resulting in longer recovery times and potential ecosystem degradation (Johnstone et al. 2016; Pausas and Keeley 2014).
- Prediction: Before reaching the fire characteristic threshold, the rate of recovery increases predictably with fire intensity; beyond this threshold, recovery becomes highly variable, indicating a tipping point that may lead to prolonged degradation and delayed regeneration (Dakos et al. 2008; Peterson 2002; van Nes et al. 2016).

Q4. Do frugivorous birds contribute to post-fire forest resilience (engineering resilience), and does this contribution depend on fire history?

- Hypothesis: Frugivores enhance forest recovery by dispersing seeds of late-successional and fleshy-fruited species, potentially accelerating regeneration in burned areas—provided that their populations are not severely reduced by fire (Bello et al. 2024; Pizo 2007; Jordano 2014).
- Prediction: Higher frugivore abundance should be associated with faster kNDVI recovery rates, particularly under low to moderate fire disturbance where frugivore-mediated seed dispersal remains functional. Under high fire severity or high recurrence, this relationship weakens or disappears because frugivore populations collapse or environmental constraints outweigh biotic dispersal effects.

Methods.

### Study area

**Fig. 1:**
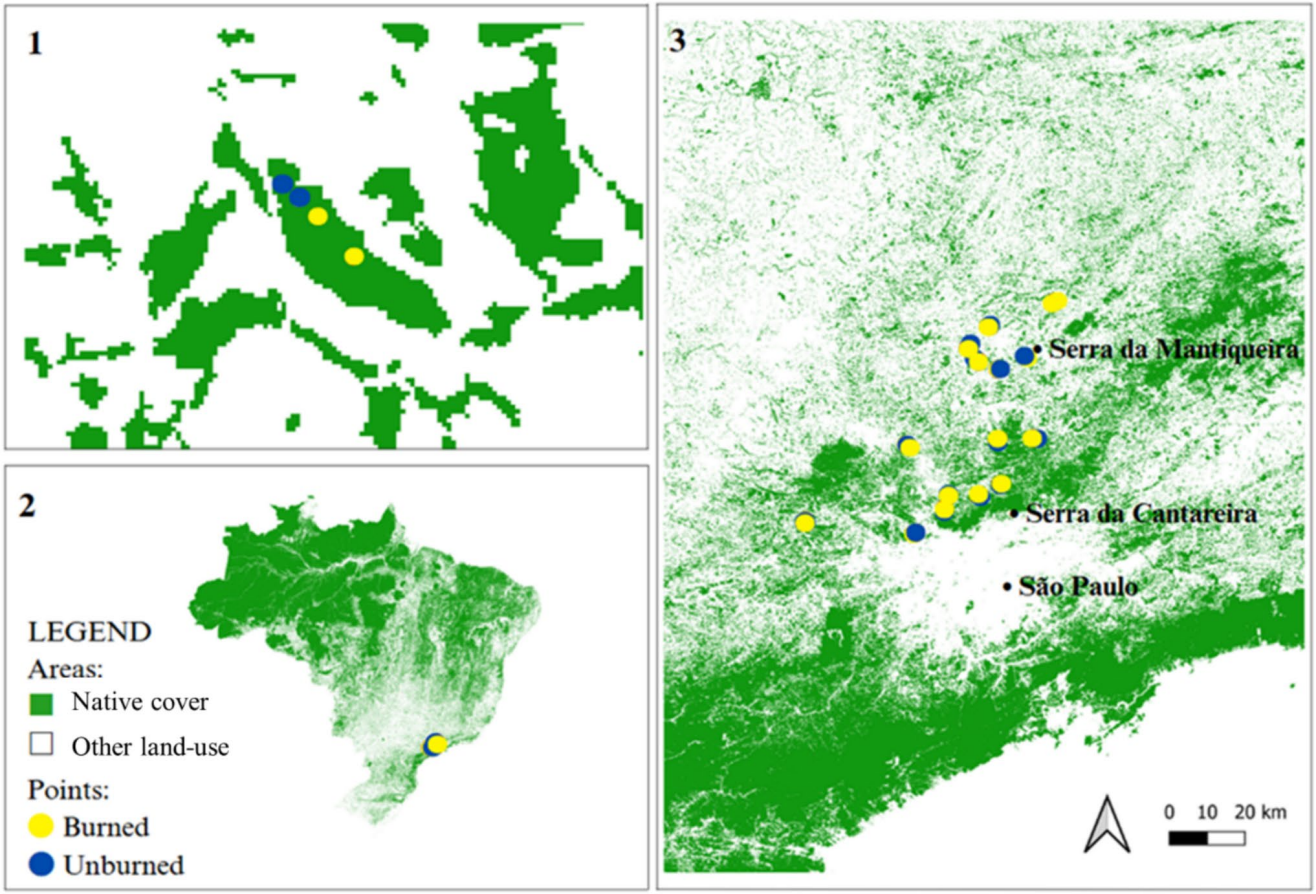
Ecological Corridor of Serra da Cantareira and Serra da Mantiqueira, located in the southeastern Atlantic Forest, Brazil, showing the distribution of sampling sites. 1 Detailed view of the local landscape structure, representing the spatial configuration of native cover and surrounding land-use types. Within each landscape, two sampling points were randomly established in burned areas and two in unburned areas. 2 General location of the study region in Brazil, highlighting the extent of native cover within the Atlantic Forest biome. 3 Spatial distribution of 30 bird sampling points used to evaluate frugivorous bird abundance across different post-fire regeneration stages. Sampling points are classified as burned (yellow) or unburned (blue) to reflect contrasting fire histories

The study was conducted in the Cantareira-Mantiqueira Corridor, a key biodiversity hotspot located in the Atlantic Forest of southeastern Brazil (Fig. 1), (Zachos et al. 2011; SEMIL, 2024). This region is recognized as a priority area for conservation because it connects two major forest remnants—the Serra da Cantareira and Serra da Mantiqueira—which are surrounded by a mosaic of small fragmented forest patches. This connectivity is crucial for maintaining species dispersal and ecological processes across the Corridor (Diniz et al. 2021).

The Cantareira-Mantiqueira Corridor is characterized by a humid subtropical climate (Cwa in the Köppen classification), with an annual mean temperature ranging from 15 °C to 22 °C and rainfall exceeding 1500 mm per year, concentrated in the summer months (Oliveira-Filho and Fontes 2000). The predominant vegetation includes montane and submontane Atlantic Forest, with a diverse composition of evergreen and semi-deciduous tree species, many of which provide using point count method resources for frugivorous birds (Marjakangas et al. 2020; Oliveira-Filho and Fontes 2000). The corridor includes a mosaic of protected areas, secondary forests, pastures, and human-modified landscapes, making it an ideal setting to assess the effects of fire on forest resilience and biodiversity (Pivello 2011; Vancine et al. 2024).

Historically, fire was not a natural component of the Atlantic Forest, as high humidity levels, high canopy closure and limited fuel availability prevented fire ignition and propagation (Pivello et al. 2021). However, land-use changes, deforestation, and climate variability have increased the incidence of human-induced fires in the region (Carvalho et al. 2022; Singh and Huang 2022). Most fire events are now anthropogenic, often originating from illegal activities, agricultural burnings, and escaped fires from nearby pastures. Recurrent fires are particularly frequent in forest edges, degraded forest patches, and areas invaded by non-native grasses, which act as fuel loads, promoting fire spread and intensification (Rezende et al. 2018).

### Sampling design

We selected 15 landscapes to assess the effects of fire on forest resilience and frugivorous bird abundance. Our selection process considered fire history, spatial distribution, and forest cover variability, ensuring a comprehensive representation of post-fire dynamics.

Each selected landscape had at least 3 hectares of burned forest and was located at least 2 km apart to reduce the spatial dependence. To capture both short-term and medium-term post-fire recovery, we selected landscapes affected by fires in 2014, 2020, and 2021. These years were chosen because they represented periods with high fire occurrence, allowing us to assess time since fire as a predictor. We delineated forest cover using Google Earth imagery (Google, 2022) and measured its extent within a 1000 m buffer around fire-affected areas. This buffer dimension was chosen based on previous evidence of landscape-scale effects on bird communities in the same region (Barros et al. 2019).

To identify and characterize fire events, we compiled fire history data from multiple remote sensing sources. We extracted fire occurrence records from INPE (National Institute for Space Research, Brazil), which detects fires using thermal radiation from satellites such as NOAA-18 and 19, METOP-B and C, TERRA and AQUA, NPP-Suomi, and NOAA-20. We analyzed LANDSAT 8 Level-2 satellite imagery from USGS Earth Explorer (2022) to detect fire scars and quantify fire severity and fire extent (see details on the section below: Predictor variables). Additionally, we incorporated MapBiomas Fire Maps for the state of São Paulo, which document fire history from 1985 to 2022.

### Predictor variables

We quantified the following variables as predictors in our models (Supporting Information—Table S1):

(1) Forest cover: We measured the proportion of unburned forested area within a 1,000 m buffer surrounding the fire-affected sites. This gradient ranged from < 11% to > 85% across landscapes.

(2) Fire frequency: We counted the number of fire events in each landscape using MapBiomas fire records, with values ranging from 1 to 11 occurrences between 1985 and 2022 (Alencar et al. 2022).

(3) Fire extent: We calculated the percentage of burned forest area within each landscape, which varied between 1.2% and 33.8%.

(4) Fire severity: We assessed fire severity using the Normalized Burn Ratio (NBR), a spectral index that identifies burned areas based on changes in vegetation reflectance. NBR relies on Near-Infrared (NIR, 0.76–0.90 µm) and Shortwave Infrared (SWIR, 2.08–2.35 µm) reflectance values.

$${\text{NBR }} = \, {{\left( {{\mathrm{NIR}} - {\mathrm{SWIR}}} \right)} \mathord{\left/ {\vphantom {{\left( {{\mathrm{NIR}} - {\mathrm{SWIR}}} \right)} {\left( {{\text{NIR }} + {\text{ SWIR}}} \right)}}} \right. \kern-0pt} {\left( {{\text{NIR }} + {\text{ SWIR}}} \right)}}$$

We obtained LANDSAT 8 Level-2 images (30-meter resolution) with minimal cloud cover from the United States Geological Survey (USGS) repository (http://earthexplorer.usgs.gov). We quantified fire severity using the Differenced Normalized Burn Ratio (dNBR), which measures changes in NBR values before and after fire events. We calculated dNBR as follows:

$${\mathrm{dNBR}} = {\mathrm{NBRpre}} - {\mathrm{fire}} - {\mathrm{NBRpost}} - {\mathrm{fire}}$$

Higher dNBR values indicated greater burn severity, associated with extensive canopy loss and ecosystem damage. In contrast, negative dNBR values identified areas where vegetation had regenerated, while near-zero values corresponded to non-vegetated surfaces such as bare soil or water bodies. Across our study landscapes, fire severity (dNBR) ranged from −0.013 to 0.17, with the highest values reflecting the most intense fire impacts.

(5) Time since fire: We identified the number of years between the most recent fire event, allowing us to assess post-fire recovery dynamics over time. The selected landscapes spanned a gradient from 1 to 8 years post-fire.

### Engineering resilience of forests

We assessed engineering resilience using time series data of the kernel Normalized Difference Vegetation Index (kNDVI) from 2014 to 2024 (Camps-Valls et al. 2021). The Normalized Difference Vegetation Index (NDVI) is a widely used vegetation index that estimates vegetation greenness and health based on reflectance in the near-infrared (NIR) and red bands. It is calculated as:

$${\mathrm{NDVI}} = {{({\mathrm{NIR}} - {\mathrm{Red}})} \mathord{\left/ {\vphantom {{({\mathrm{NIR}} - {\mathrm{Red}})} {({\mathrm{NIR}} + {\mathrm{Red}})}}} \right. \kern-0pt} {({\mathrm{NIR}} + {\mathrm{Red}})}}$$

However, NDVI often suffers from saturation effects in areas with dense vegetation, limiting its ability to detect changes in highly productive forests. To overcome this limitation, the kNDVI index was developed using a tangent transformation of the squared NDVI, which improves robustness to noise and enhances sensitivity across spatial and temporal gradients. As a result, kNDVI shows a stronger correlation with field-based measurements of primary productivity and is more effective at capturing subtle shifts in vegetation dynamics (Camps-Valls et al. 2021; Wang et al. 2023).

We derived kNDVI from Landsat 8 imagery at 30-m resolution and 16-day composite, masking pixels affected by clouds and cloud shadows. To estimate engineering resilience, we analyzed the temporal trend of kNDVI using the Mann–Kendall test, a non-parametric approach for detecting monotonic trends in time series data (Camps-Valls et al. 2021).

In this context, engineering resilience is defined as the time required for the forest to recover after fire disturbance, measured by the rate and direction of kNDVI change over time. A positive and highly inclined trend in kNDVI indicates a faster recovery, suggesting that the ecosystem is regaining vegetation productivity efficiently (Caughlin et al. 2020). Conversely, a negative or neutral trend reflects delayed or inhibited recovery, potentially signaling persistent degradation or environmental constraints on post-fire regeneration (Supporting Information—Table S1).

### Frugivorous bird community sampling

We conducted bird sampling using the point count method (Sutherland 2006), which provides precise estimates of species abundance while controlling for environmental variables and observation bias. This method is particularly useful in heterogeneous ecosystems.

In each landscape, we randomly established two sampling points in burned forest and two in unburned forest patches. To minimize repeated counts, we maintained a minimum distance of 200-m between the points. At each point, we recorded all birds observed or heard within a 50-m radius over a 20-min period. Sampling occurred between 5:30 am and 10:00 am, coinciding with peak bird activity. We surveyed each landscape twice on different days, ensuring a total sampling effort of 40 min per point. The sampling took place between October 2022 and January 2023, covering the reproductive period of frugivorous birds. We classified birds as frugivores based on Wilman et al. (2014), considering species whose diet consists of at least 50% fruit consumption (Supporting Information—Table S1).

To account for the hierarchical structure of our sampling design, we considered the two points within the same forest patch type (i.e., burned or unburned) as subsamples and treated species abundances at these points as repeated measures. As a result, each sampling unit was composed of four (two points surveyed twice), leading to a total of 30 sample units across all landscapes (Sampling effort = 80 min per point) (Fig. 1).

To account for imperfect detection in our repeated count data, we estimated species abundances using hierarchical N-mixture models (Royle 2004) implemented in the R package unmarked (functions unmarkedFramePCount and pcount; Fiske and Chandler 2011; Kellner et al. 2023). In these models, the latent abundance at each sampling point is treated as a random variable, and the observed counts from repeated surveys are modeled as binomial outcomes conditional on true abundance and detection probability. We used four repeated point counts per sampling point as temporal replicates, allowing us to separate variation in detectability from variation in abundance across burned and unburned forest patches (Benoit et al. 2018).

For each frugivorous bird species, we fitted an N-mixture model with landscape–habitat as a site covariate on abundance and constant detection. We then predicted abundance for each species and sampling point and summed predicted abundances across all frugivore species within each sampling unit (Supporting Information—Table S2). To improve the reliability of estimates, we retained only species with predicted abundance ≥ 1 individual and detection probability ≥ 0.1, following Dorazio and Royle (2005) Royle (2004). This threshold minimized biases associated with rare detections and strengthened the robustness of abundance estimates.

### Statistical analysis

#### Data preprocessing

All numerical predictor variables were standardized using *z*-score transformation to improve model convergence and facilitate interpretation. Standardization ensures that all predictors have a mean of 0 and a standard deviation of 1, preventing variables with large numerical ranges from dominating the model and improving numerical stability.

Multicollinearity among predictors was assessed using pairwise Pearson correlations. Time since fire was excluded from all analyses due to strong collinearity with other fire metrics (|*r*|> 0.7) (Dormann et al. 2013).

#### Assessing data distribution

We evaluated the distribution of both response variables—frugivore abundance and forest resilience (kNDVI trend)—by fitting Gaussian, Gamma and Beta probability distributions and comparing Akaike Information Criterion (AIC) values (Akaike 1974). Gaussian errors provided the best compromise between goodness of fit and model simplicity for both variables. Consequently, all models were fitted using Gaussian error structures with identity links. Residual diagnostics were visually inspected to confirm this choice.

Modeling strategy aligned with the research questions.

*Q1* + *Q2. Modeling the effects of fire characteristics and habitat loss on frugivore abundance*. To evaluate how fire characteristics influence frugivore abundance and whether these effects depend on habitat loss, we modeled frugivore abundance using the full dataset, which includes both burned and unburned forest patches. Unburned sites are essential for this analysis because they represent the baseline variation in frugivore communities across different forest conditions, ensuring that fire effects are not confounded with natural differences in assemblages.

We first fitted a set of Generalized Linear Mixed Models (GLMMs) using the glmmTMB package (Brooks et al. 2017), incorporating landscape as a random effect. The random-effect variance for landscape was extremely small (≪ 0.001) and did not improve model fit, indicating negligible landscape-level variation in the responses. We therefore proceeded with Generalized Linear Models (GLMs), which provided a more parsimonious and interpretable framework. We compared additive, interactive and non-linear (second-order polynomial) formulations of fire and landscape predictors.

For each fire attribute (severity, frequency, extent), we compared:

(1) Additive effects: frugivore abundance ~ forest cover + fire metric.

(2) Interactive effects (buffering or amplification): frugivore abundance ~ forest cover × fire metric.

(3) Nonlinear effects: frugivore abundance ~ forest cover + fire metric + (fire metric)^2^

Candidate models were restricted to a maximum of two predictors (plus interaction or quadratic terms) to maintain statistical power given the sample size. Including more than two predictors (plus interaction or quadratic terms) would violate recommended sample–parameter ratios for reliable inference and greatly increase model uncertainty (Burnham and Anderson 2002). We therefore restricted all candidate models to a maximum of two predictors, ensuring stability of parameter estimates and interpretability. Model selection followed an information-theoretic approach based on ΔAICc < 2 and model weights > 0.10 (MuMIn). Residual diagnostics were assessed using DHARMa, and multicollinearity verified via VIF.

This analytical framework allowed us to test:

1. Direct effects of fire characteristics on frugivore abundance;
2. Context-dependent effects, i.e., whether forest cover modifies fire impacts.

*Q3. Does forest recovery decline nonlinearly beyond a fire threshold?* For forest resilience analyses, we used only burned forest patches, because unburned sites do not exhibit a recovery trajectory and including them would artificially inflate variance in fire-history predictors. Thus, all models of kNDVI Trend were restricted to burned sites, ensuring that resilience metrics reflect only post-fire vegetation dynamics.

We modeled kNDVI Trend using GLMs with both linear and quadratic terms for fire severity, fire extent, and fire frequency. Nonlinear terms (e.g., fire frequency^2^) tested for curvature consistent with ecological thresholds.

To further evaluate the presence of abrupt shifts, we fitted segmented (piecewise) regressions relating kNDVI Trend to fire frequency, estimating breakpoints directly from the data using the segmented package. Breakpoints were interpreted as potential fire-frequency thresholds beyond which recovery declines or becomes highly variable. Model selection followed the same AICc-based criteria as for Q1–Q2.

Q4. Do frugivorous birds contribute to post-fire forest resilience, and does this contribution depend on fire history?

To test whether frugivores influence vegetation recovery, we included frugivore abundance as a predictor of kNDVI Trend, using only burned forest sites, where resilience can be meaningfully measured. We fitted the following complementary models:

1. Direct frugivore effect: kNDVI Trend∼frugivore abundance
2. Joint effects of fire and frugivores: kNDVI Trend∼fire metric + frugivore abundance
3. Nonlinear fire effects + frugivores: kNDVI Trend∼fire metric + (fire metric)^2^ + frugivore abundance

We also tested interaction terms (e.g., fire metric × frugivore abundance), but none were supported by AICc criteria. Across models, residual structure and VIF values confirmed good fit and low collinearity. This set of models allowed us to evaluate whether:

- 1) Frugivores directly enhance forest recovery;
- 2) Their effects persist after accounting for fire history;
- 3) Frugivores modulate fire impacts on resilience.

## Results

We recorded a total of 35 frugivorous bird species, representing 12 different families. The families with the highest species richness were Thraupidae (9 species), Psittacidae (5 species), and Cotingidae (4 species) (Supplementary material Table S2).

The most abundant species overall included *Turdus rufiventris* and *Thraupis sayaca*, both recorded in burned and unburned forests. However, some species were restricted to unburned forests, including *Amazona amazonica, Diopsittaca nobilis, Elaenia spectabilis, Euphonia violacea, Neopelma chrysolophum, Ramphastos dicolorus*, and *Tangara cyanoventris*. In contrast, species with the highest estimated abundance in burned forests were *Turdus rufiventris, Thraupis sayaca, Turdus amaurochalinus, Brotogeris chiriri,* and *Patagioenas cayennensis.*

**Fig. 2:**
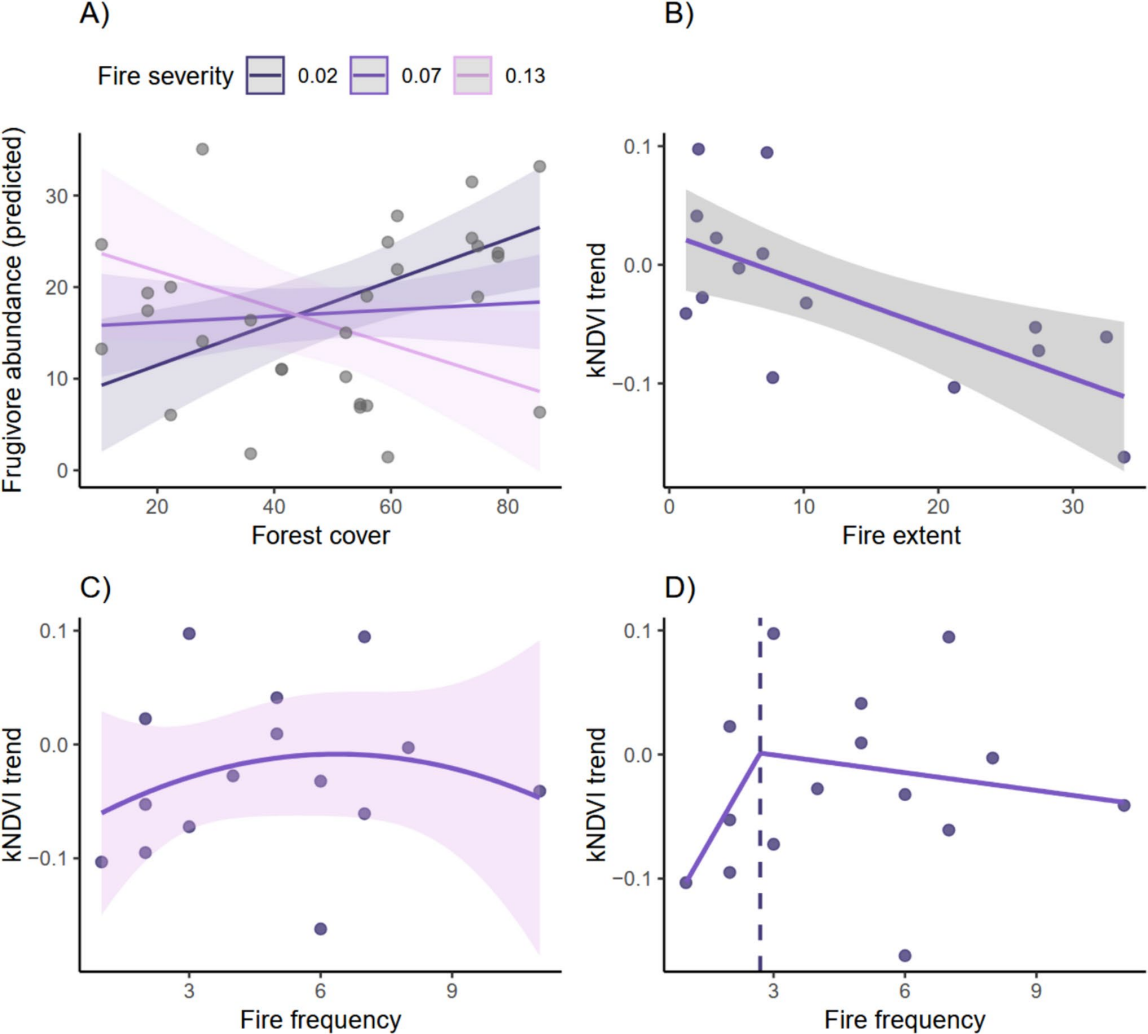
Relationships between fire characteristics, landscape structure, and ecological responses derived from generalized linear models. **A** Effect of the interaction between forest cover and fire severity on frugivore abundance, showing how the direction and magnitude of the response depend on burn intensity. **B** Linear negative effect of fire extent on forest resilience (kNDVI trend). **C** Nonlinear (quadratic) relationship between fire frequency and kNDVI trend, indicating increasing variability at higher fire recurrence. **D** Segmented regression identifying an estimated fire-frequency breakpoint (~ 3 events), beyond which forest resilience becomes more variable and declines more slowly. Shaded areas indicate 95% confidence intervals for model predictions

Across all fire metrics (severity, frequency, extent), additive models revealed that frugivore abundance was not explained by fire attributes alone (Table 1). In contrast, we found a clear interaction between fire severity and forest cover influencing frugivore abundance (Table 1; Fig. 2A). The direction of the effect of forest cover varied across the fire‐severity gradient: at low fire severity, frugivore abundance increased with forest cover, whereas at high fire severity the relationship reversed, and landscape sites with higher forest cover supported fewer frugivores than sites with lower cover.

**Table 1:** Results of generalized linear models evaluating the effects of fire characteristics and forest cover on frugivore abundance and forest resilience measured by kNDVI trend. Models include additive, interactive, nonlinear (quadratic), and segmented formulations depending on the ecological hypothesis tested. The table presents unstandardized coefficient estimates with 95% confidence intervals in parentheses. Significant predictors (*p* < 0.05) are shown in bold. For linear and quadratic models, *R*^2^ and adjusted *R*^2^ values are reported; for the segmented model, the estimated breakpoint is also provided.

Predictors	Frugivore_abundance		kNDVI_trend		kNDVI_trend		kNDVI_trend		kNDVI_trend	
	Estimates	*p*	Estimates	*p*	Estimates	*p*	Estimates	*p*	Estimates	*p*
(Intercept)	3.46	0.5	0.03	0.2	−0.03	0.2	−0.16	0	−0.03 ^*^	**0.04**
	(−7.13–14.04)		(−0.02–0.07)		(−0.06–0.01)		(−0.51–0.18)		(−0.05–−0.00)	
Fire severity	171.63 ^*^	**0**								
	(37.29–305.97)									
Forest cover	0.31 ^**^	**0**								
	(0.12–0.50)									
Fire severity × forest	−3.91 ^**^	**0**								
Cover	(−6.39–−1.43)									
Fire extent			−0.004 ^***^	**0**					−0.05^***^	** < 0.001**
			(−0.006–−0.002)						(−0.08–−0.03)	
Fire frequency [1st					0.03	0.7				
degree]					(−0.12 – 0.18)					
Fire frequency [2nd					−0.06	0.4				
degree]					(−0.21 – 0.09)					
Fire frequency							0.06	0		
							(−0.13–0.25)			
U1 fire frequency							−0.07	0		
							(−0.26–0.13)			
Psi1 fire frequency							0	1		
							(−3.28–3.28)			
Frugivore abundance									−0.01	0.28
									(−0.04–0.01)	
Observations	30		15		15		15		15	
R^2^/R^2^ adjusted	0.290/0.176		0.463		0.059		0.132/−0.105		0.502/0.366	

* *p* < 0.05 ** *p* < 0.01 *** *p* < 0.001

Values in bold indicate statistically significant results (*p* < 0.05)

Neither forest cover nor fire severity alone explained frugivore abundance in the absence of the interaction. Interactions between forest cover and other fire attributes (frequency, extent) were evaluated but were not supported relative to the severity × cover model. This context-dependent response indicates that forest cover only buffers fire impacts under low-severity conditions, while this buffering effect collapses under high severity.

Forest resilience, quantified as kNDVI trend, was strongly associated with fire history. Fire extent had a clear negative linear effect (Table 1), with larger burned areas showing slower recovery (Fig. 2B). Fire frequency exhibited a nonlinear pattern, although statistical support for the quadratic term was weak (Table 1). Still, the fitted curve suggested:

- (1) Recovery tended to vary little across low-to-intermediate fire frequencies, with broad and overlapping confidence intervals indicating high uncertainty;
- (2) At higher recurrence levels, kNDVI trend values became more dispersed, consistent with increasingly variable recovery outcomes (Fig. 2C).

Segmented regression estimated a breakpoint at approximately three fire events (Fig. 2D), but uncertainty around this estimate was high, and the breakpoint term lacked statistical support. The pattern nonetheless suggests a tendency toward reduced predictability of recovery beyond this recurrence level.

Contrary to expectations, frugivore abundance showed no detectable effect on kNDVI trend (Table 1). Although frugivore abundance was included in the best-supported fire-extent model (ΔAICc < 0.1), its coefficient was weak and close to zero, indicating that model support was driven almost entirely by the strong influence of fire extent. Thus, short-term post-fire forest resilience in these landscapes appears shaped primarily by fire history rather than by frugivore-mediated processes such as seed dispersal.

## Discussion

### Fire, habitat loss, and frugivore abundance

Frugivore responses to fire were strongly context-dependent, emerging only when landscape structure (forest cover) was considered jointly with fire severity. Fire severity, forest cover, and other fire metrics alone showed no detectable effects on frugivore abundance, but their interaction revealed a marked pattern: forest cover enhanced frugivore abundance only under low-severity conditions. However, under high fire severity, the effect of forest cover reversed, such that forests with higher cover supported fewer frugivores than those with lower cover. This inversion indicates that the buffering role of forest cover collapses under severe disturbances, likely due to extensive canopy loss, reduced fruiting substrates, and diminished structural complexity (Barlow et al. 2002; Morales et al. 2023).

These findings support the hypothesis that fire and habitat loss jointly shape frugivore populations, and highlight the need to evaluate these disturbances in combination rather than isolation. Similar interactive effects have been reported in tropical and subtropical forests, where fire-induced structural simplification disproportionately affects species dependent on canopy resources, fruit availability, and vertical habitat heterogeneity (Barlow et al. 2002; Barlow and Peres 2004a, b; Haugaasen et al. 2003; Morales et al. 2023).

This pattern is consistent with ecological mechanisms described in previous studies: high-severity fires typically cause extensive tree mortality, reducing fruiting individuals and nesting substrates, which tend to disproportionately affect specialist frugivores. In contrast, low-severity burns often leave the canopy relatively intact, maintaining habitat heterogeneity that supports frugivore persistence (Keeley 2009).

Forest cover is a well-established predictor of frugivore abundance (Hasui et al. 2024; Hutto et al. 2020; Menezes et al. 2016). However, our results demonstrate that forest cover alone cannot offset the effects of severe fires. Even intact remnants may lose their capacity to sustain frugivore populations under high-severity disturbance, underscoring the vulnerability of fragmented tropical landscapes exposed to increasingly intense fires (Renner et al. 2024).

### Fire characteristics and engineering resilience

Forest resilience, measured by kNDVI Trend, was strongly shaped by both the extent and recurrence of fire, but in distinct ways. Fire extent imposed a clear linear constraint on recovery: as burned area increased, regeneration declined proportionally. This pattern is consistent with known mechanisms such as greater isolation from seed sources, intensified edge desiccation, and harsher microclimatic exposure in large burn scars, all of which slow post-fire vegetation regrowth (Donato et al. 2009; Harvey et al. 2016).

Fire frequency, in contrast, produced a more complex, nonlinear response. Although statistical support for the quadratic term was limited, both the fitted curve and the segmented regression revealed a general pattern in which recovery showed modest changes at low to intermediate recurrence but became increasingly variable at higher frequencies. Beyond approximately three fire events, kNDVI values displayed marked dispersion, suggesting that ecosystems become more sensitive to fine-scale environmental conditions and less able to recover consistently across sites.

This growing variability aligns with ecological expectations of cumulative degradation: repeated burns can deplete seed banks, inhibit trees from reaching reproductive maturity, and shift competitive dynamics in ways that diminish regeneration capacity (Stevens-Rumann and Morgan 2016; Braziunas et al. 2023). The emergence of an estimated breakpoint around three fire events is consistent with fire ecology frameworks that describe how frequent fires can push ecosystems toward tipping points or transitions into alternative, degraded states (Ross et al. 2024).

### Fire-vegetation feedbacks

Our findings support the existence of positive fire–vegetation feedbacks, in which recurrent fires promote vegetation states that become progressively more flammable and less capable of re-establishing canopy structure. Repeated burning often favors fire-adapted grasses and fast-growing species that dry rapidly and accumulate surface fuels, thereby intensifying subsequent fires and reinforcing the cycle (Pausas and Keeley 2019). As these processes unfold, post-fire trajectories begin to diverge: in some sites, residual seed banks or favorable microclimatic conditions allow partial recovery, whereas in others, repeated burns inhibit woody regeneration and drive transitions toward grass-dominated or arrested-succession states. This increasing divergence helps explain the greater dispersion of kNDVI Trend values observed at high fire recurrence levels, reflecting a growing dependence on fine-scale environmental conditions (Pivello et al. 2021). Such patterns underscore that resilience is shaped not only by disturbance intensity but also by self-reinforcing interactions between vegetation and fire behavior.

### Weak direct effects between frugivore abundance and forest recovery

Contrary to our expectations, frugivore abundance did not meaningfully influence forest recovery, even though it appeared in the top-ranked statistical model. Its effect size was extremely small, and the model’s explanatory power was driven almost entirely by the strong negative effect of fire extent. These findings indicate that, during the first decade after fire, forest recovery is governed primarily by the disturbance regime—especially the size and recurrence of burned areas—rather than by seed-dispersal processes mediated by frugivores.

Such weak biotic influence is consistent with the ecological mechanisms that dominate early successional stages in tropical forests. Post-fire environments are characterized by reduced canopy cover, elevated temperatures, lower moisture retention, and frequent soil degradation, all of which impose strong abiotic constraints on seedling establishment regardless of seed availability (Tepley et al. 2018; Bello et al. 2024). Under these conditions, regeneration tends to be driven by stress-tolerant pioneer species, many of which rely on wind dispersal or small generalist dispersers, not on specialized vertebrate frugivores (Tabarelli and Peres 2002).

This pattern aligns with classic successional theory: in the first stages of forest regeneration, plant communities are dominated by small-seeded species that colonize rapidly through abiotic dispersal pathways, while the contribution of vertebrate frugivores increases only gradually as forest structure recovers (Tabarelli and Peres 2002). Only in later successional phases—when canopy shading, microclimatic buffering, and organic matter accumulation have been re-established—do larger-seeded species that depend on medium- and large-bodied frugivores begin to regenerate successfully (Metzger and Brancalion 2016; Vidal et al. 2014).

Thus, although frugivores remain essential for long-term forest dynamics, their immediate influence on post-fire resilience is expected to be limited. In severely altered landscapes, fire-driven environmental constraints overshadow biotic processes, delaying the role of frugivores until more advanced successional stages when environmental filters weaken and dispersal limitation becomes more relevant (Robinson et al. 2014; Bello et al. 2024).

### Limitations and future directions

While our study provides valuable insights into fire-driven ecological dynamics, some limitations should be considered:

1. Time since fire—Strong collinearity with fire extent prevented inclusion in modeling. A broader temporal gradient is necessary to disentangle the roles of fire age and fire extension.
2. Fire frequency threshold—Although segmented regression suggests a threshold near three events, model support for the quadratic term was limited. Larger sample sizes would improve the robustness of threshold detection.
3. Long-term frugivore effects—Our dataset captures early–mid recovery stages (< 10 years). Frugivore-mediated recovery may become more evident in later successional stages.
4. Other potential drivers—Soil properties, drought stress, dispersal limitation from surrounding landscapes, and species-specific regeneration traits likely interact with fire signals. Incorporating multi-scale environmental and biological variables would strengthen predictive models.

### Practical implications for fire management and forest restoration

Our findings have important implications for fire management and restoration efforts in tropical forests:

1. *High-frequency and high-extent burns require active restoration*—Passive regeneration is unlikely to be sufficient when disturbance exceeds local resilience limits.
2. *Forest cover remains important but insufficient under high severity—*Preventing extreme fires is crucial to maintain the buffering function of forest cover*.*
3. *Preventing recurrent fires is crucial for maintaining resilience*—Because resilience drops sharply beyond three fire events, preventing repeated burns is key to avoiding irreversible ecosystem transitions.
4. *Incorporating resilience thresholds into policy*—The ~ 3-fire threshold may help guide land-use planning, fire suppression priorities, and restoration investment.

## Conclusion

Fire and habitat loss interact synergistically to shape frugivore populations. Forest cover buffers fire effects under low severity but loses this protective role under high severity, leading to sharp declines in frugivore abundance. Contrary to initial expectations, frugivores exerted no detectable short-term influence on forest recovery, which was instead governed by fire extent and nonlinear responses to recurrent burning. A threshold near three fire events emerged as a critical limit beyond which recovery becomes highly variable and unpredictable. These findings highlight the importance of integrating fire management with habitat conservation to prevent transitions to degraded, fire-susceptible states and maintain long-term tropical forest resilience.

## Supplementary Information

Below is the link to the electronic supplementary material.

Supplementary file1 (XLSX 31 KB)

## Data Availability

The data supporting the findings of this study are available in the Supplementary Material (Tables S1 and S2).
